# Expression of CIP2A in renal cell carcinomas correlates with tumour invasion, metastasis and patients’ survival

**DOI:** 10.1038/bjc.2011.492

**Published:** 2011-11-10

**Authors:** J Ren, W Li, L Yan, W Jiao, S Tian, D Li, Y Tang, G Gu, H Liu, Z Xu

**Affiliations:** 1 Department of Urology, Qilu Hospital, Shandong University, 107# Wenhua Xi Road, Jinan 250012, PRC; 2 The key Laboratory of Cardiovascular Remodeling and Function Research, Chinese Ministry of Education and Chinese Ministry of Public Health, Department of Cardiology, Qilu Hospital, Shandong University, 107# Wenhua Xi Road, Jinan 250012, PRC; 3 Department of Microbiology/Key Laboratory for Experimental Teratology of Chinese Ministry of Education, School of Medicine, Shandong University, 44# Wenhua Xi Road, Jinan 250012, PRC

**Keywords:** CIP2A, RCC, metastasis, prognosis, survival

## Abstract

**Background:**

Cancerous inhibitor of protein phosphatase 2A (CIP2A) drives cellular transformation. The objective of this study was to detect the potential effects of CIP2A in renal cell carcinomas (RCCs).

**Methods:**

A total of 107 RCC patients were involved in the study. Cancerous inhibitor of protein phosphatase 2A expression was investigated by real-time PCR and immunohistochemistry. *In vitro*, we examined the expression of CIP2A and c-Myc and tested the migration and invasion capability of A498 and KRC/Y cells with scratch migration assay and Matrigel invasion assay after down-regulating CIP2A expression using siRNA.

**Results:**

Cancerous inhibitor of protein phosphatase 2A was over-expressed in RCC tissues. Clear cell RCC showed an even higher-CIP2A expression level than papillary or chromophobe RCC did. The CIP2A immunostaining level was positively correlated with primary tumour stage, lymph node metastasis, distant metastasis, TNM stage and histological grade (all *P*<0.05). High-CIP2A expression implied poor survival for patients (*P*<0.05). Cancerous inhibitor of protein phosphatase 2A depletion by siRNA down-regulated c-Myc expression and attenuated the migration and invasion of RCC cells.

**Conclusion:**

Higher-CIP2A expression positively correlates with the aggressive phenotype of RCCs, and predicts poor prognosis for patients. Cancerous inhibitor of protein phosphatase 2A may be a novel target for prevention and treatment of RCC metastasis and recurrence.

Renal cell carcinoma (RCC) is the most common carcinoma of the adult kidney, and its incidence has gradually increased during the last decades (Eble *et al*, 2004). Resection of the diseased kidney is a standard therapeutic approach for RCC. However, ∼30% of patients develop metastatic disease after surgery (Zisman *et al*, 2002), and median survival of those patients is only about 13 months (Cohen and McGovern, 2005). The driving factors underlying RCC metastasis remain poorly defined and better understanding of RCC metastasis mechanisms is required for the development of rational strategies for the prevention and treatment of RCC recurrence.

Cancerous inhibitor of protein phosphatase 2A (CIP2A), a cellular protein phosphatase 2A (PP2A) inhibitor, promotes the stability of c-Myc protein by inducing c-Myc serine 62 (S62) phosphorylation and inhibiting its degradation mediated by PP2A (Junttila *et al*, 2007). Experimentally identified as an oncoprotein, CIP2A contributes to immortalisation and malignant transformation of human cells. Importantly, recent studies show that CIP2A is over-expressed in various human malignancies including head and neck squamous cell carcinoma (Junttila *et al*, 2007), oral squamous cell carcinoma (Basile and Czerninski, 2010), oesophageal squamous cell carcinoma (Qu *et al*, 2010), colon (Junttila *et al*, 2007), gastric (Li *et al*, 2008), breast (Come *et al*, 2009), prostate (Vaarala *et al*, 2010), tongue (Bockelman *et al*, 2011), lung (Dong *et al*, 2011; Ma *et al*, 2011), cervical cancer (Liu *et al*, 2011) and acute myeloid leukaemia (Wang *et al*, 2011). Cancerous inhibitor of protein phosphatase 2A has also been found to be a prognostic factor for patients with gastric cancer (Khanna *et al*, 2009), early-stage tongue cancer (Bockelman *et al*, 2011) and non-small cell lung cancer (Dong *et al*, 2011).

Expression and biological function of CIP2A in RCC has so far not been investigated. In the present study, we determined CIP2A expression in RCC and explored the potential effects of CIP2A on RCC metastasis and patients’ survival.

## Patients and Methods

### Patients and tissue specimens

One hundred and seven patients with RCC (86 clear cell RCC, 14 papillary RCC and 7 chromophobe RCC) and 6 patients with renal hamartoma were included in the study, which was approved by the local ethics committee. The patients underwent radical nephrectomy at Qilu Hospital, Shandong University, between 2004 and 2011. None of the patients had received chemotherapy or radiotherapy before surgery. The diagnosis was confirmed by histopathological examination of the specimens. After surgery, tumour specimens, corresponding tumour adjacent renal tissues and normal renal tissues from patients with renal hamartoma were collected and stored in liquid nitrogen until use. Parts of each sample were fixed in formalin, embedded in paraffin and stored in the Department of Pathology, Qilu Hospital. All the patients were staged according to the tumour node metastasis staging system (Eble *et al*, 2004) and nuclear grade was evaluated on the basis of the Fuhrman criteria (Fuhrman *et al*, 1982). Clinical data of all the patients were collected from hospitalisation and subsequent records. Detailed information is listed in Table 1. Follow-up of 85 patients was done, and 47 of them were alive at the end of the follow-up.

### Cell lines and culture conditions

Human RCC cell lines A498 (ATCC, Manassas, VA, USA), KRC/Y (kindly provided by Dr D Xu at Karolinska Institute, Sweden) were cultured at 37°C, 5% CO_2_ in RPMI 1640 (Invitrogen, Carlsbad, CA, USA) containing 10% FBS (Invitrogen), 100 U ml^–1^ penicillin (Sigma, St Louis, MO, USA) and 100 *μ*g ml^–1^ streptomycin (Sigma).

### Small interfering RNA treatment

The chemically modified siRNA targeting CIP2A and control siRNA were purchased from Invitrogen. The sequence of siRNA for CIP2A was 5′-GACAACUGUCAAG UGUACCACUCUU-3′. Cells were transfected with either CIP2A or control siRNA using Lipofectamine 2000 (Invitrogen) according to the manufacturer's instructions.

### RNA extraction, reverse transcription PCR and real-time quantitative PCR

Total RNA was extracted from tissue specimens and cells with different treatment using TRIzol (Invitrogen) according to the manufacturer's instructions. A total of 1 *μ*g RNA was used for reverse transcription with M-MLV reverse transcriptase (Fermentas, Burlington, Canada). The PCR primers used in the study were CIP2A: 5′-CCATATGC TCACTCAGATGATGT-3′ (forward) and 5′-GTGTATCATCTCCA CAGAGAGTT-3′ (reverse) and *β*-actin: 5′-AGTTGCGTTACACCCTTTCTTG-3′ (forward) and 5′-CAC CTTCACCGTTCCAGTTTT-3′ (reverse). Quantitative real-time polymerase chain reaction (QPCR) was performed using SYBR Green PCR Master Mix (Applied Biosystems, Foster City, CA, USA) in a 7000 Real-Time PCR System (Applied Biosystems). *β*-Actin was used as the reference gene. Ct values of the samples were calculated, and the relative levels of CIP2A mRNA were analysed by the 2^−ΔΔCt^ method.

### Immunohistochemistry

In all, 5-*μ*m paraffin sections were prepared for the experiments. De-pariffinisation was carried out with xylene and rehydrated, and endogenous peroxidase activity was quenched by 3% hydrogen peroxide in methanol. Antigen retrieval was done by boiling in a microwave oven in 10 mM citrate buffer (pH 6.0). Block non-specific binding was performed with 5% bovine serum albumin, and the slides were incubated with anti-CIP2A antibody (ab84547, Abcam, Cambridge, UK; 1 : 150 dilution). After washing, the slides were incubated with horseradish peroxidase-conjugated goat anti-rabbit IgG. Colour was developed with DAB Horseradish Peroxidase Color Development Kit (P0202, Beyotime, Haimen, China).

The slides were scored by two independent pathologists, who were blind to the data of the patients, according to the intensity and percentage of CIP2A staining. Cancerous inhibitor of protein phosphatase 2A expression was scored as 0 (negative), 1 (mild, 0–4% positive cells), 2 (moderate, 5–49% positive cells) and 3 (strong, ⩾50% positive cells).

### Western blot

Total cellular protein was extracted with a lysis buffer containing 50 mM Tris–HCl, 150 mM NaCl, 1 mM PMSF, 1 mM EDTA, 1% Triton X-100, 1% NaTDC, 0.1% SDS, pH 7.4 (DBI Bioscience, Shanghai, China). Protein concentration was quantified using BCA Protein Quantitative Kit (DBI Bioscience). And then 25 *μ*g protein was resolved by SDS–PAGE, and transferred to PVDF membrane (Millipore, Billerica, MA, USA). The PVDF membranes were blocked with 5% skim milk and then probed with the primary antibody CIP2A (ab61863, Abcam), c-Myc and *β*-actin (Santa Cruz Biotechnologies, Santa Cruz, CA, USA). Then, the membranes were incubated with anti-mouse IgG and developed with the enhanced chemiluminescent Kit (DBI Bioscience).

### Colony formation assay

A498 and KRC/Y cells were transfected with control or CIP2A siRNA for 72 h. Then, the cells were planted into six-well plate (300 cells per well) and incubated for 10 days, as described (Li *et al*, 2010). Plates were stained with Giemsa, and positive colony formation (with >50 cells) was counted.

### Scratch migration assay

A498 and KRC/Y cells were transfected with control or CIP2A siRNA for 24 h. Thereafter, the cells were scratched with the 1000-*μ*l pipette tips. Plates were washed twice with PBS in order to remove the detached cells, and incubated using the complete growth medium. Wound closure (cells migrating into the scratched empty space) was observed and measured after 24 and 48 h.

### Matrigel invasion assay

Cell invasion was performed at a 24-well Transwell chamber with a pore size of 8 *μ*m (Costar, NewYork, NY, USA). The insert was coated with 50 *μ*l Matrigel (dilution at 1 : 2; BD Bioscience, Franklin Lakes, NJ, USA). A498 and KRC/Y cells were trypsinised after transfection with control or CIP2A siRNA for 72 h and transferred to the upper Matrigel chamber in 100 *μ*l of serum free medium containing 1 × 10^5^ cells and incubated for 24 h. The lower chamber was filled with medium supplemented with 10% FBS as chemoattractants. The membranes were fixed and stained using 0.1% crystal violet. The numbers of invaded cells were counted in five randomly selected high-power fields ( × 400) under a microscope.

### Statistical analysis

Real-time PCR data were analysed using the Mann–Whitney test. Associations between CIP2A immunostaining level and clinicopathological parameters were analysed using the *χ*^2^-test. The data of migration and invasion were analysed with the Student's *t*-test. Univariate association with survival was evaluated using Kaplan–Meier curves, and tested by Log-Rank test (SPSS version 16.0 for Mac; SPSS, Inc., Chicago, IL, USA). Multivariate analyses were performed according to Cox proportional hazards regression model. *P*-value <0.05 was considered to be statistically significant.

## Results

### Expression of CIP2A and clinicopathological variables of RCC patients

**Figure 1:**
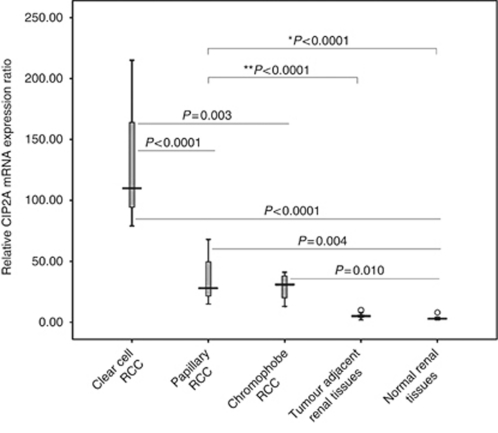
CIP2A mRNA expression in RCCs and non-neoplastic renal tissues. ^*^RCC specimens *vs* normal renal tissues. ^**^RCC specimens *vs* tumour adjacent renal tissues. Clear cell RCC *vs* papillary RCC (*P*<0.0001) and clear cell RCC *vs* chromophobe RCC (*P*=0.003). Papillary RCC *vs* chromophobe RCC (*P*=0.636). Clear cell RCC *vs* normal (*P*<0.0001). Papillary RCC *vs* normal (*P*=0.004). Chromophobe RCC *vs* normal (*P*=0.010). Tumour adjacent renal tissues *vs* normal renal tissues (*P*=0.107). Error bars: the range of each group's data.

Expression of CIP2A mRNA was first assessed using real-time quantitative PCR in 26 RCC specimens (15 clear cell RCC, 7 papillary RCC and 4 chromophobe RCC), 15 corresponding adjacent tissues and 6 normal renal tissues. Cancerous inhibitor of protein phosphatase 2A was expressed at higher levels in RCC tissues (89.58±11.81) than in matched adjacent tissues (5.27±0.56) and normal renal tissues (3.67±0.92) (both *P*<0.0001). Intriguingly, CIP2A expression in clear cell RCC specimens (130.67±11.48) was significantly higher than that in papillary RCC (36.14±7.71) and chromophobe RCC (29.00±6.06) (Figure 1). The levels of CIP2A mRNA expression in clear cell RCC, papillary RCC and chromophobe RCC were higher than that in normal renal tissues (all *P*<0.05) (Figure 1).

**Figure 2:**
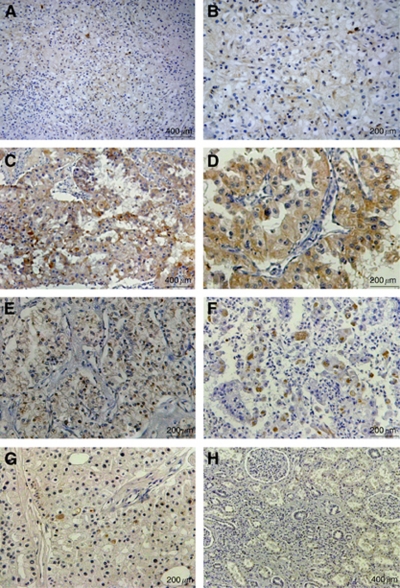
Immunohistochemical staining of CIP2A in RCC specimens and normal renal tissues. CIP2A immunohistochemical staining was performed on different types of RCCs and normal renal tissues, and representative images are shown. (**A** and **B**) Weak staining of CIP2A in clear cell RCC (**A**: magnification, × 100; **B**: magnification, × 200). (**C** and **D**) Strong staining of CIP2A in clear cell RCC (**C**: magnification, × 100; **D**: magnification, × 200). (**E**) CIP2A staining in the cytoplasm and nucleus of clear cell RCC (magnification, × 200). (**F**) CIP2A staining in papillary RCC (magnification, × 200). (**G**) CIP2A staining in chromophobe RCC (magnification, × 200). (**H**) Extremely weak or diffuse CIP2A staining in renal tubules of normal renal tissues (magnification, × 100).

We further analysed CIP2A protein level in 107 RCC tissues, 19 tumour adjacent tissues and 6 normal renal tissues using an immunohistochemical approach. Consistent with CIP2A mRNA expression profile, the presence of the CIP2A protein was found in 75 of 107 (70%) of cancer samples, whereas only 6 of 19 (32%) tumour adjacent tissues and 1 of 6 (17%) normal renal tissues exhibited weak or diffuse CIP2A expression (*P*<0.0001). Cancerous inhibitor of protein phosphatase 2A staining was mainly observed in the cytoplasm and occasionally found in the nucleus of tumour cells (Figure 2A–E). Only weak or diffuse CIP2A staining was observed in some renal tubules in normal renal tissues (Figure 2H). Furthermore, the CIP2A staining level significantly correlated with primary tumour stage, lymph node metastasis, distant metastasis, TNM stage and histological grade (all *P*<0.05). There was no significant association between CIP2A expression and patients’ gender and age (Table 1).

### CIP2A expression and postoperative survival of RCC patients

**Figure 3:**
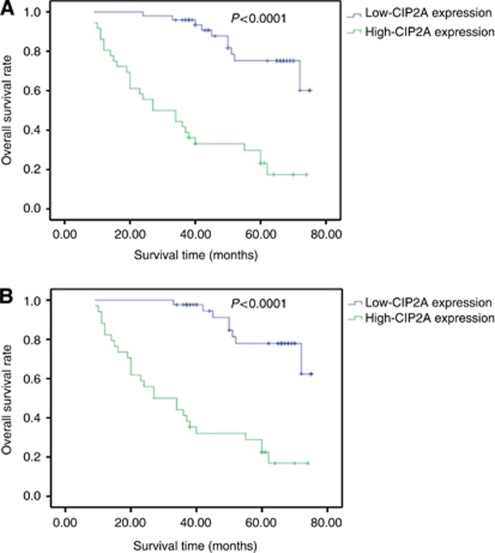
Kaplan–Meier curves of OS according to CIP2A expression. Patients with high-CIP2A expression have an overall lower survival rate than patients with low-CIP2A expression. (**A**) Patients of the three histological types (*n*=85). (**B**) Patients of clear cell RCC (*n*=76) (Log-Rank test, both *P*<0.0001).

Overall survival (OS) was used for survival analysis. Death caused by RCC was appointed as the end point of analysis. Overall survival was defined as the time interval between surgery and death. Univariate 5-year OS revealed that patients with advanced primary tumour stage, lymph nodes metastasis, distant metastasis and high histological grade had worse outcomes (Table 2). The OS rate of patients with high-CIP2A expression (staining intensity: 2–3) was significantly lower than that with low-CIP2A expression (staining intensity: 0–1) (*P*<0.0001; Figure 3A). In addition, multivariate analysis indicated that advanced primary tumour stage, distant metastasis and high-CIP2A expression were independent prognostic factors for RCC patients (Table 3).

Among clear cell RCC patients, univariate and multivariate analysis showed analogous results (Tables 2 and 3). Also, higher-CIP2A expression indicated poor prognosis (*P*<0.0001; Figure 3B) and high-CIP2A expression was a significantly independent prognostic factor for clear cell RCC patients (*P*=0.001; Table 3).

### Effects of CIP2A depletion on c-Myc expression, cell migration and invasion *in vitro*

**Figure 4:**
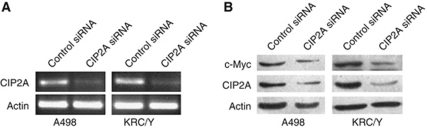
Depletion of CIP2A down-regulates c-Myc protein expression in renal cancer cells. (**A**) RT–PCR analysis of CIP2A mRNA expression in A498 and KRC/Y cells transfected with the specific siRNA targeting CIP2A for 72 h. (**B**) Western blot analysis of CIP2A and c-Myc protein expression in A498 and KRC/Y cells transfected with the CIP2A siRNA for 72 h. Efficient depletion of CIP2A expression was verified.

**Figure 5:**
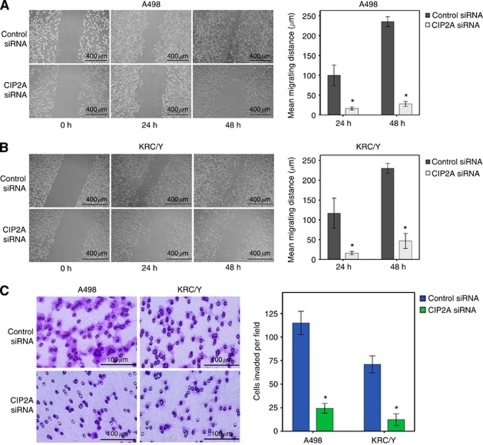
CIP2A depletion attenuated the migration and invasion capability of RCC cells *in vitro*. (**A** and **B**) Scratch migration assay of A498 and KRC/Y cells, respectively. The wound closure was measured in photographs at 24 and 48 h. (Left) Representative photographs of scratch migration assay. (Right) Quantification of relative migrating distances of cells with indicated treatment. (**C**) Matrigel invasion assay of A498 and KRC/Y cells. (Left) Representative images of invading cells treated with indicated siRNA. (Right) Quantification of relative numbers of invading cells representing average counts from five fields of view. Error bars: standard error of the mean (s.e.m.). ^*^P<0.05.

Cancerous inhibitor of protein phosphatase 2A mRNA and protein expression was remarkably inhibited in A498 and KRC/Y cells treated with specific siRNA for CIP2A compared with those treated with control siRNA (Figure 4A and B). Furthermore, depletion of CIP2A by siRNA resulted in inhibition of c-Myc protein expression in both A498 and KRC/Y cells (Figure 4B). A498 and KRC/Y renal cancer cells were transfected with CIP2A siRNA and scratched using a Pipette tip 24 h after transfection. The migration capability of A498 and KRC/Y transfected with CIP2A siRNA was markedly inhibited at 24 h (A498 control *vs* CIP2A siRNA: 100±11 *vs* 16±2, *P*<0.0001; KRC/Y control *vs* CIP2A siRNA : 117±15 *vs* 16±2, *P*<0.0001) and 48 h (A498 control *vs* CIP2A siRNA: 235±5 *vs* 28±3, *P*<0.0001; KRC/Y control *vs* CIP2A siRNA: 230±5 *vs* 47±8, *P*<0.0001) after scratch (Figure 5A and B). In addition, Matrigel invasion assay demonstrated that the number of invasive cells passing through the filter were significantly reduced after knocking down of CIP2A in A498 (control *vs* CIP2A siRNA: 115±5 *vs* 24±2, *P*<0.0001) and KRC/Y cells (control *vs* CIP2A siRNA: 71±4 *vs* 12±3, *P*<0.0001) (Figure 5C).

We further examined whether cell proliferation capacity was altered in cells with transfection of CIP2A siRNA. However, it was found that CIP2A depletion did not have a measurable blocking effect on cell proliferation in colony formation assay (data not shown).

## Discussion

The present study provides the first evidence that CIP2A over-expression widely occurs in RCCs and positively correlates with advanced disease stages and metastasis, and negatively affects patients’ OS. Consistent with these clinical findings, experiments on RCC cell lines demonstrate that CIP2A depletion significantly inhibits migration and invasion of these RCC cells *in vitro*. Collectively, these results indicate a critical role of CIP2A in driving disease progression and spread of RCCs.

Previous studies have shown that aberrant expression of CIP2A is associated with progressive diseases in a number of other human malignancies including breast (Come *et al*, 2009), tongue (Bockelman *et al*, 2011), lung (Dong *et al*, 2011; Ma *et al*, 2011), gastric (Li *et al*, 2008), head and neck cancers (Junttila *et al*, 2007) and chronic myeloid leukaemia (Lucas *et al*, 2011). More recently, CIP2A expression has been found to be associated with synovial hyperplasia and invasive function of fibroblast-like synoviocytes in rheumatoid arthritis (Lee *et al*, 2011). It is currently unclear how CIP2A promotes tumour cell invasion and metastasis. Based on the role of CIP2A in stabilising and up-regulating the MYC oncoprotein, it is likely that MYC is involved in CIP2A-stimulated invasiveness of tumour cells. The MYC oncoprotein is capable of conferring a selective advantage on cancer cells by stimulating proliferation, cell survival, differentiation blockade, genetic instability and angiogenesis, all of which may contribute to metastasis (Baudino *et al*, 2002; von Rahden *et al*, 2006; Gordan *et al*, 2007; Guffei *et al*, 2007; Vaque *et al*, 2008). Furthermore, MYC is necessary for the invasion and metastasis of cancer cells in experimental xenografts independent of its effects on proliferation and survival (Wolfer *et al*, 2010). More recently, MYC has been found to regulate the epithelial-to-mesenchymal transition, a required cellular programme for invasion and migration. MYC achieves this by stimulating TGF-*β*-mediated activation of the SNAIL transcription factor through a microRNA network involving the LIN28B/let-7/HMGA2 pathway (Smith *et al*, 2009; Viswanathan *et al*, 2009; Khew-Goodall and Goodall, 2010; Ma *et al*, 2010; Helland *et al*, 2011). All these effects of MYC may contribute to tumour cell metastasis driven by CIP2A, and further study is required to elucidate the role of MYC in CIP2A-mediated RCC metastasis.

In some types of tumour cells, CIP2A depletion leads to impaired proliferation potential largely due to diminished MYC expression (Li *et al*, 2008; Come *et al*, 2009; Dong *et al*, 2011). However, neither A498 nor KRC/Y cells exhibited significant alterations in colony formation after CIP2A was knocked down. Clearly, CIP2A inhibition-mediated attenuation of RCC cell invasion is independent of cellular proliferation. This suggests that other factors may be involved in CIP2A promotion of RCC metastasis in addition to MYC.

Cancer metastasis can be one of the key factors affecting patients’ survival, but other elements may also lead to poor survival. Recent studies demonstrate that CIP2A displays many other activities, for instance, promoting stem cell self-renewal and giving cancer cells resistance to chemotherapeutic agents (Chen *et al*, 2010; Kerosuo *et al*, 2010; Choi *et al*, 2011). Increased self-renewal of cancer stem cells and drug resistance may lead to treatment failure, and thereby poor outcomes for patients. Moreover, CIP2A was also implicated in the blockade of cellular senescence and differentiation, as shown in our previous study (Li *et al*, 2008). Therefore, different mechanisms result in unfavourable prognosis in RCC patients with CIP2A over-expression.

Little is known about the mechanism underlying the aberrant expression of CIP2A in malignant cells. In gastric cancer, *Helicobacter pylori* infection up-regulates CIP2A expression through the Src and ERK pathways (Zhao *et al*, 2010). Cancerous inhibitor of protein phosphatase 2A is up-regulated by human papillomavirus 16 E7 oncoprotein in cervical cancer (Liu *et al*, 2011). In addition, CIP2A and MYC form a positive feedback regulation loop to affect each other's expression (Khanna *et al*, 2009). It is currently unclear what contributes to CIP2A over-expression in RCCs. In the present study, it was found that clear cell RCC exhibited enhanced CIP2A expression at a higher frequency. It is well established that VHL mutation and subsequent HIF1/2 *α* dysregulation play a key part in the pathogenesis of clear cell RCC (Krieg *et al*, 2000; Krishnamachary *et al*, 2006); therefore, it may be interesting to probe the effects of these molecular events on CIP2A expression. Also, differences in CIP2A distribution between normal renal tissues and RCC tissues were noticed, and the nuclear accumulation of CIP2A was occasionally seen in RCC tissues. Based on the above observations, dysregulation of CIP2A may occur at different levels in RCCs.

In conclusion, it was found that CIP2A is over-expressed in RCCs especially in clear cell RCC and plays an important role in RCC metastasis. Higher expression of CIP2A positively correlates with the aggressive phenotype of RCCs, and predicts poor outcome of patients. Therefore, CIP2A may be a novel target for prevention and treatment of RCC metastasis and recurrence.

## Figures and Tables

**Table 1:** Characteristics of patients with RCC and associations between CIP2A expression and clinicopathologic variables

**Variable**	**Total**	**CIP2A staining**		***P*-value**
		**0–1**	**2–3**	
*Gender*				
Male	79	55	24	0.125
Female	28	15	13	
				
*Age, years (median 55)*				
<55	44	33	11	0.082
⩾55	63	37	26	
				
*T stage*				
T_1,2_	74	58	16	<0.0001
T_3,4_	33	12	21	
				
*N stage*				
N_0_	93	68	25	<0.0001
N_1,2_	14	2	12	
				
*M stage*				
M_0_	96	69	27	<0.0001
M_1_	11	1	10	
				
*TNM stage*				
I–II	66	58	8	<0.0001
III–IV	41	12	29	
				
*Histological grade*				
G_1,2_	61	47	14	0.004
G_3,4_	46	23	23	
				
*Histology type*				
Clear cell	86	51	35	0.022
Papillary	14	12	2	
Chromophobe	7	7	0	

Abbreviations: CIP2A=cancerous inhibitor of protein phosphatase 2A; RCC=renal cell carcinoma.

0–1: low-CIP2A expression; 2–3: high-CIP2A expression.

**Table 2:** Univariate 5-year overall survival of RCC patients (*n*=85)

**Parameters**	**Patients of the three histological types (*n*=85)**			**Patients of clear cell RCC (*n*=76)**		
	**Total**	**5-Year overall survival**	***P*-value**	**Total**	**5-Year overall survival**	***P*-value**
*T stage*						
T_1,2_	57	73.68%	<0.0001	48	75.00%	<0.0001
T_3,4_	28	17.86%		28	17.86%	
						
*N stage*						
N_0_	72	62.50%	<0.0001	64	60.94%	0.001
N_1,2_	13	15.38%		12	16.67%	
						
*M stage*						
M_0_	76	61.84%	<0.0001	68	60.29%	<0.0001
M_1_	9	0		8	0	
						
*Histological grade*						
G_1,2_	50	72.00%	<0.0001	47	70.21%	<0.0001
G_3,4_	35	31.43%		29	27.59%	
						
*CIP2A staining*						
0–1	49	79.59%	<0.0001	42	81.00%	<0.0001
2–3	36	22.22%		34	20.60%	

Abbreviations: CIP2A=cancerous inhibitor of protein phosphatase 2A; RCC=renal cell carcinoma.

**Table 3:** Cox regression analysis in predicting the overall survival of RCC patients

	**Patients of the three histologic types (*n*=85)**			**Patients of clear cell RCC (*n*=76)**		
**Risk factors**	**OR**	**95% CI**	***P*-value**	**OR**	**95% CI**	***P*-value**
*Primary tumour stage*						
T_1,2_	2.788	1.277–6.087	0.010	3.403	1.453–7.967	0.005
T_3,4_						
						
*Distant metastasis*						
M_0_	3.743	1.525–9.187	0.004	3.066	1.226–7.668	0.017
M_1_						
						
*Histological grade*						
G_1,2_	1.616	0.750–3.486	0.220	1.028	0.454–2.326	0.948
G_3,4_						
						
*CIP2A staining*						
0–1	1.771	1.041–3.013	0.004	4.607	1.884–11.267	0.001
2–3						

Abbreviations: CIP2A=cancerous inhibitor of protein phosphatase 2A; OR=odds ratio; RCC=renal cell carcinoma; 95% CI=95% confidence interval.
